# A Paradoxical Clinical Coincidence: Benign Paroxysmal Positional Vertigo and Bilateral Vestibulopathy

**DOI:** 10.3390/jcm12103413

**Published:** 2023-05-11

**Authors:** Nicolás Pérez-Fernández, Sara Saez Coronado, Cristina Zulueta-Santos, Fernando Neria Serrano, Jorge Rey-Martinez, Melisa Blanco, Raquel Manrique-Huarte

**Affiliations:** 1 Department of Otorhinolaryngology, Marquesado de Santa Marta 1, Clínica Universidad de Navarra, 28047 Madrid, Spain; czulueta@unav.es (C.Z.-S.); melimelo15@gmail.com (M.B.); 2 Department of Otorhinolaryngology, Clínica Universidad de Navarra, 31008 Pamplona, Spain; rmanrique@unav.es; 3 Department of Otorhinolaryngology, Hospital Universitario de Móstoles, 28938 Madrid, Spain; sarasaezcoronado@gmail.com; 4 Department of Otorhinolaryngology, Facultad de Medicina, Universidad Francisco de Vitoria, 28223 Madrid, Spain; fernando.neria@ufv.es; 5 Department of Otorhinolaryngology, Hospital Universitario Donostia, 20014 San Sebastian, Spain; bendermh@hotmail.com

**Keywords:** Vestíbulo-ocular reflex, dizziness, vestibulopathy

## Abstract

Benign paroxysmal positional vertigo (BPPV) and bilateral vestibulopathy (BVL) are two completely different forms of vestibular disorder that occasionally occur in the same patient. We conducted a retrospective review searching for that coincidence in our database of the patients seen over a 15-year period and found this disorder in 23 patients, that is 0.4%. More frequently they occurred sequentially (10/23) and BPPV was diagnosed first. Simultaneous presentation occurred in 9/23 patients. It was subsequently studied, but in a prospective manner, in patients with BPPV on all of whom a video head impulse test was performed to search for bilateral vestibular loss; we found it was slightly more frequent (6/405). Both disorders were treated accordingly, and it was found that the results follow the general trend in patients with only one of those disorders.

## 1. Introduction

The major categories of vestibular symptoms are vertigo, dizziness, vestibular-visual and postural. All were recently arranged in a taxonomy based on triggers and situational occurrence broad enough to cover most of what is clinically recorded during anamnesis, being purely phenomenological and avoiding topo-diagnostic implications [1]. 

Benign paroxysmal positional vertigo (BPPV) is an episodic vestibular syndrome triggered by head motion when performing positional changes. It is one of the most common diagnoses at outpatient clinics, and its frequency depends on the clinical context or specialty [2]. The criteria for its correct diagnosis have recently been delineated [3]. Vertigo elicited during positional maneuvers (Dix-Hallpike, supine head-hanging, or, in supine head roll to right or left) marks the suspicion that demands demonstration during the vestibular examination of coincidental nystagmus [4]. The pathophysiological explanation lies in the existence of otoconial debris that floats in the endolymph and moves to the affected semicircular canal or remains adhered to the cupula. Most cases occur as a primary and idiopathic disease with a rapid good response to treatment which means a very straightforward clinical procedure [5]. However, BPPV can occur as a secondary or comorbid disorder as in Ménière’s disease, which is a type of disease in which vertigo occurs without any trigger: in 14% of the patients with definite Ménière’s disease, some of the vertigo crises will be diagnosed as typical BPPV and treated accordingly [6]. Positional vertigo is also another form of clinical presentation in vestibular migraine [7]. Additionally, both disorders (Ménière’s and migraine) share an important overlap in which positional vertigo or nystagmus must be considered [8,9].

This finding is not restricted to BPPV and MD or Vestibular migraines. Previous authors have considered that different vestibular disorders are highly interrelated in 3.7% of the patients who receive at least two diagnoses to better define their clinical status [10].

Bilateral vestibulopathy or bilateral vestibular loss (BVL) is an untriggered chronic vestibular syndrome and, contrary to BPPV, is infrequent, representing less than 5% of the diagnosis at specialized clinics [11]. Recently, clear diagnostic criteria have also been laid down for BVL [12]. This is a differential diagnosis to be considered in any patient with chronic instability (which may become more severe when visual information is reduced, such as walking in darkness) and oscillopsia or movement-induced blurred vision. The abnormal ocular response during the right- and left-ward head-thrust test at the bedside [13] will be the red flag to pursue in the differential diagnosis of this entity [14]. In BVL there is an abrupt or progressive decline in the function of the vestibular periphery at the level of the receptor in the inner ear or the nerve; it can also occur after a period of vertigo crises and be associated with neurological symptoms. BVL shares some links with disorders such as vestibular migraine and Ménière’s disease. Recurrent vertigo is also mentioned by 33% of the patients with BVL but it is not clear whether this is spontaneous or positional [15] and is more frequent (43%) in patients with dissociated BVL which indicates that the amount of vestibular deficit in one side is clearly different from that measured on the other side with any of the methods for vestibular testing commonly used. This form of presentation of the disorder represents 20.8% of BVL and, in that series, it is interesting to note that there was also one case diagnosed with BPPV [16]. This association (BPPV and BVL) had already been reported in four patients of a series of 240 patients with BPPV all of whom suffered from posterior semicircular canalolithiasis; in those, BVL was due to gentamicin-induced vestibular ototoxicity [17]. In a later and broader analysis of patients with BPPV affecting the posterior semicircular canal, BVL was found in 21/2847 patients who were considered to have a secondary form of BPPV [18].

The study aimed to analyze the prevalence of both disorders in a retrospective and prospective study. We were interested in not restricting the diagnosis of BPPV to posterior semicircular canalithiasis alone and in the amount of vestibular asymmetry found in these patients.

## 2. Material and Methods

This is a review work of the experience at one institution at two different centers (Pamplona and Madrid). The information in this paper pertaining to BVL was previously published but in a broader study on bilateral vestibulopathy [11]; no data on BPPV were mentioned in that paper. We present the results of a retrospective study in a single institution to address how frequently both disorders occur and of a prospective study to analyze how frequent BVL is when searched for systematically in patients with BPPV. Patients were seen between 1 September 2006 and 31 August 2021. This spans 15 years of work by three experienced neurotologists working part-time and full-time at one (RMH) or both (NPF) centers. All patients gave informed consent to the use of their correctly anonymized clinical data for research purposes.

### 2.1. Bedside Testing

This was carried out on all patients and included ocular motility (saccades and smooth-pursuit), spontaneous nystagmus with and without visual fixation (the latter with Frenzel goggles), the post-head-shake nystagmus or the skull-vibration induced nystagmus; positional tests: head-hanging or hyperextension in a supine position, head-roll to right and left in a supine position, and the Dix-Hallpike test to the right and left sides. Other tests were performed in accordance with clinical characteristics or ongoing findings during examination.

### 2.2. Bilateral Vestibulopathy

As this study was carried out partially with patients seen in a period before the current criteria were published [11], we checked that they all shared the same clinical characteristics: criteria A and B of the guidelines (Table 1). Regarding vestibular testing, some were diagnosed because of findings in the caloric and rotatory chair testing while others (those after 2011) were in the vHIT alone. The caloric test has, for years, been the gold standard for vestibular testing, and the rotatory chair test is the recommended evaluation to confirm BVL. Recently, the video head-impulse test (vHIT) has become the preferred evaluation both at the bedside and in the laboratory to test for vestibular function and allow the testing of the vertical canals, which was not possible with the beforementioned tests [19].

Caloric test. The test was performed using two-channel VNG equipment (Ulmer VNG, v. 1.4 (SYNAPSYS, Marseille). Each ear was irrigated with water at different temperatures (44 °C and 30 °C). The induced nystagmus response was characterized by its slow-phase velocity during the duration of recording (2 min) or until it faded away. In this study, all patients diagnosed with BVL had a reflectivity or sum of the responses from both ears and each irrigation (4 stimuli) below 15 °s^−1^. This result was first confirmed with the ice water test in which when using water at 4 °C no response was obtained from each ear [20].

**Figure 1:**
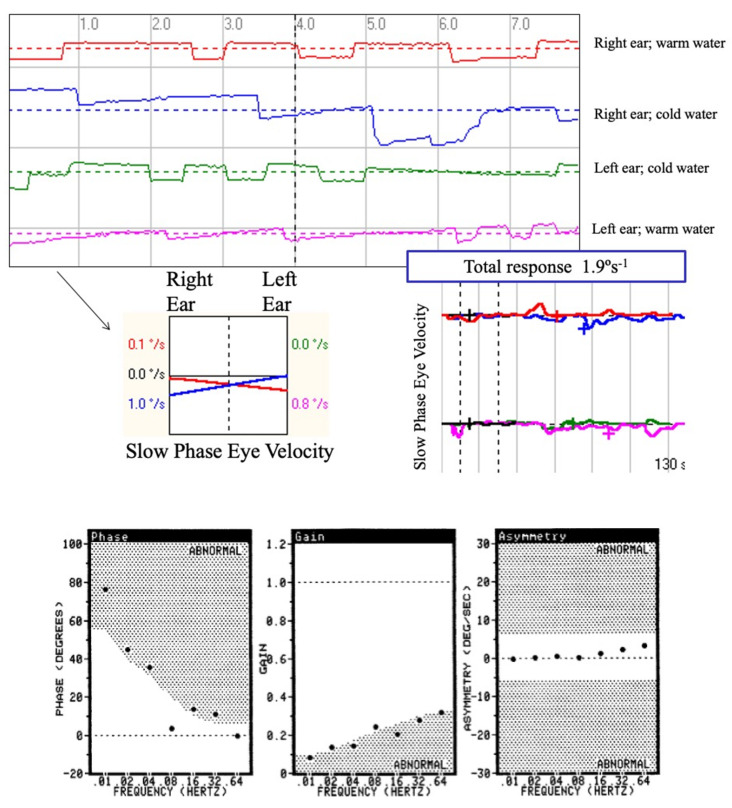
Vestibular test results in a patient diagnosed with bilateral vestibulopathy due to systemic gentamicin treatment. A: caloric test representation which shows a bilateral vestibular areflexia as no nystagmus response is obtained after stimulating each ear with cold and warm water and which has been confirmed in the ice water test in which no response was obtained from stimulating both ears. B: rotatory chair test which shows low gain and phase lag in the sinusoidal harmonic acceleration test.

Rotatory chair test. This was performed using a CHARTR-RVT system (ICS Medical Corporation, Schaumburg, IL, USA). The rotatory chair is housed in an enclosure to perform the test in the dark. The head is positioned so that both horizontal canals are close to the plane of stimulus (i.e., at the gravitational horizontal). In the first impulsive rotational test, the patient was subjected to velocity steps to the right and left. The velocity steps involved the patient undergoing an angular acceleration of 100 s^−1^ for 1 s, rotation at a constant velocity for 60 s, and finally a deceleration to 0 s^−1^ within 1 s. This procedure was performed three times in a clockwise direction and three times in a counterclockwise direction, and the TC for each was averaged (TCave). In the sinusoidal harmonic acceleration test (SHA), the individual was subjected to sinusoidal oscillation about a yaw axis at various frequencies (0.01, 0.02, 0.04, 0.08, 0.16, 0.32, and 0.64 Hz), with a peak angular velocity of 50 s^−1^. From the chair velocity and slow-phase eye velocity, two parameters of the VOR were calculated: phase and gain. To confirm the findings of bilateral reduced response in the caloric test, the TCave had to be below 8 s and the SHA test, gain and phase had to be significantly lower than normal in at least three consecutive frequencies (Figure 1).

**Figure 2:**
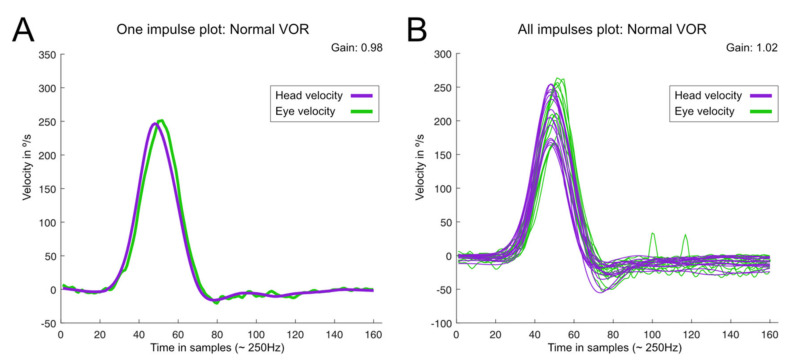
Graphical representation of a normal result in the video head impulse test (vHIT). (**A**): the result of a single head impulse; (**B**): the result of several impulses. In the former, the gain of the VOR was 0.98 and in the latter, the mean gain of the VOR was 1.02. This figure was obtained using a specific open-access program https://github.com/bendermh/HITCal (accessed on 9 March 2023) [23].

**Figure 3:**
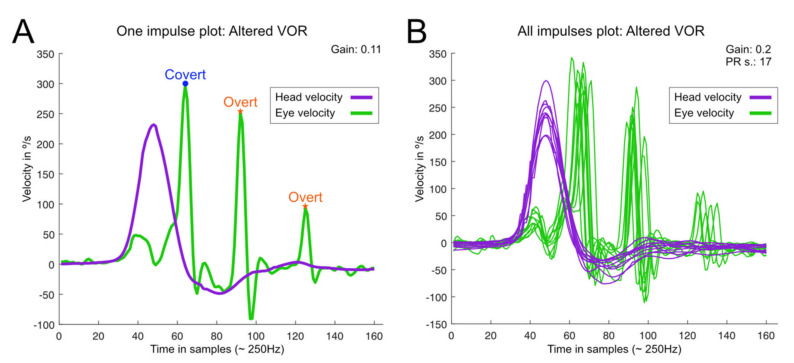
Graphical representation of an abnormal video head-impulse test result in a patient with bilateral vestibulopathy. This is the patient in Figure 1, and the analysis of the right horizontal canal stimulation is shown as an example and was performed with HITCAL software. The data shown correspond to horizontal rightward head impulses. (**A**): the result of a single head impulse; (**B**): the result of several impulses. In the former, the gain of the VOR was 0.11 and in the latter, the mean gain of the VOR was 0.2. In both corrective refixation saccades are shown and classified as covert (during the head impulse) and overt (once the head is stopped). This figure was obtained using a specific program obtained in open access https://github.com/bendermh/HITCal (accessed on 9 March 2023) [23].

**Figure 4:**
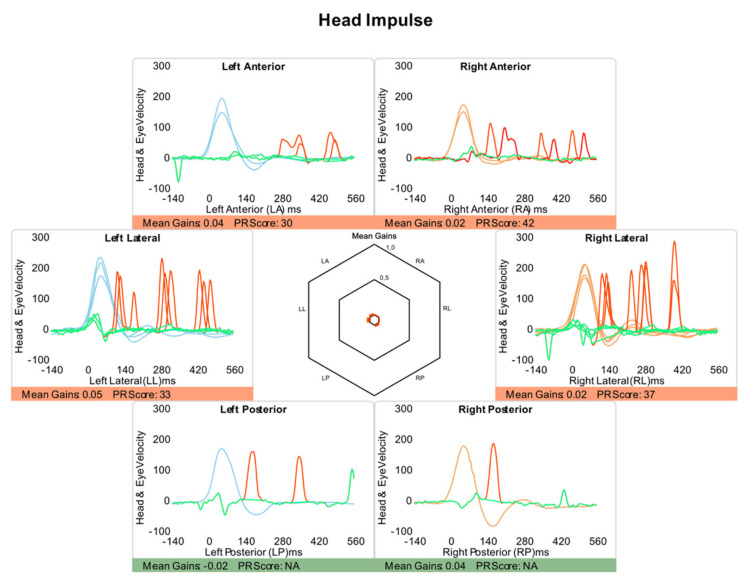
Result in the video head-impulse test of a patient with a posttraumatic BVL. This figure was obtained using a specific open-access program https://github.com/bendermh/HITCal (accessed on 9 March 2023) [23].

Video-Head Impulse Test. This was performed using a vHIT system that allows the testing of all six semicircular canals (GN Otometrics, Denmark). For this test, the patient wears a pair of lightweight, tightly fitting goggles on which are mounted a small video camera and a mirror that reflects the image of the patient’s right eye into the camera. The eye is illuminated by a low-level infra-red light-emitting diode. A small sensor on the goggles measures the head movement. Calibration is performed and the procedure of vestibulo-ocular testing is initiated. Horizontal semicircular canals (right, RC, and, left, LC) are tested first: the clinician asks the patient to keep staring at an earth-fixed target 1 m. in front and gives the patient brief, abrupt, head rotations through a small angle (about 10–20 degrees), unpredictably turning to the left or right on each trial. At the end of each head turn, the head-velocity stimulus and eye-velocity response are displayed simultaneously on the screen. In a full test, at least 10 impulses are delivered randomly in each direction. The first pair of vertical canals is then evaluated: the patient’s head is rotated 30° to the right while staring at the same earth-fixed target as before, but now out of the corner of his/her eye. Brief, abrupt, forward, and backward head impulses are then carried out, which allow stimulation of the left superior semicircular canal (LA) and the right posterior semicircular canal (RP) respectively. After a full test of at least 10 impulses in each direction, the second pair of vertical canals is evaluated: for this, the patient’s head is rotated 30° to the left while staring at the earth-fixed target. Now, forward head impulses stimulate the right superior semicircular canal (RA) and backward impulses stimulate the left posterior semicircular canal (LP). A full test of at least 10 impulses in each direction is performed. At the end of each full test, all the head velocity stimuli and eye velocity responses are displayed on the computer screen, together with a graph of the calculated VOR gain (ratio of eye velocity to head velocity) for every head rotation (Figure 2). The gain was evaluated as normal or abnormal according to norms by age [21,22]. The second parameter is re-fixation saccades (RS) classified as covert and overt, the first being re-fixation saccades that take place during the cephalic impulse and the second taking place when the impulse has ended. A test was considered normal when the gain of the VOR was according to expected results consistent with age and there were no refixation saccades in any of the six canals evaluated. A test was abnormal when at least in the plane of 1 canal the response is lower in gain than expected and there are re-fixation saccades (Figure 3). In this study, in all the patients with BVL, the response was abnormal for the stimulation of the horizontal right and left superior, horizontal, and posterior semicircular canals as shown in the patient in Figure 4, and the gain in the horizontals was below 0.6.

### 2.3. Benign Paroxysmal Positional Vertigo

Patients were seen for recurrent short (<1 min) attacks of vertigo induced by lying down, waking up, or turning over in the supine position, and certain position changes while performing activities during the day; this symptom was reproduced during testing and the duration of the attack was usually <1 min except in the case of horizontal canal involvement which was usually longer. In all cases under Frenzel goggles, nystagmus was elicited (after some latency) in the Dix-Hallpike test, supine roll test, or in head-hanging; in each, its characteristics will indicate (1) the side and canal affected (posterior, horizontal, or superior) and, (2) the mechanism: cupulolithiasis or canalithiasis. The patients were treated accordingly with the proper particle repositioning maneuver (PRM) and seen 30–60 min later to confirm resolution, persistence, or migration of the otoconia; in the latter two situations, the patient was treated again. All patients were seen 10–15 days later until the resolution of the BPPV.

In this case, only a vestibular bedside examination was done and, when considered by the neurotologist in charge, vHIT also. At one of the centers, vHIT was performed on all BPPV-diagnosed patients before the corresponding particle repositioning maneuver.

### 2.4. Inclusion Criteria

To be included in this work, after clinical and laboratory diagnosis several follow-ups were required to confirm the diagnosis in the case of BVP. In the case of BPPV patients, they were followed until the resolution of the initial symptom.

### 2.5. Statistical Analysis

For descriptive purposes, qualitative data are represented as absolute (*n*) and relative (%) frequency; for quantitative variables, data are shown as mean ± SD or mean (95% CI).

For demographic analysis, differences between normally distributed data were assessed with the t-student test or the one-way ANOVA test, and for non-normally distributed data the Wilcoxon test or the Kruskal–Wallis test was performed. Differences between percentages were determined by using Fisher’s exact test.

All analyses were performed using R software v 4.1 (R Foundation for Statistical Computing, Vienna, Austria). A *p*-value < 0.05 was considered statistically significant.

## 3. Results

In the period of study, we were able to include in our database 5562 patients of which 3533 (63.6%) were female and 2029 (36.4%) male. Of them, 405 are part of a prospective study in which of all patients diagnosed with BPPV the vHIT was performed.

Of the total number of patients in 239 (4.3%) BVL was the main diagnosis and in 2297 (41.3%) BPPV. The mean number of follow-ups in the former was 2.3 and the number of PRM performed until resolution was 4179, which makes 1.9 PRM per patient.

Both diagnoses (BVL&BPPV) and the interest of this paper occurred in 23 patients, 0.4% of the total number of patients, and represents 10% of the patients with BVL and 1% of those with BPPV. They were 10 (43.5%) male and 13 (56.5%) female. Their mean age was 75 years [95% confidence interval (CI95): 64–82]. In 10 patients the first diagnosis was BPPV, in 4 BVL and, in 9 both were simultaneously done. When BPPV was first diagnosed the second diagnosis (BVL) was done 51 months (CI95 32–108) after and, when BVL was first diagnosed the second diagnosis (BPPV) was done 18 months (CI95 12–29) after. Those periods of time between diagnoses were significantly different (*p* = 0.034).

**Figure 5:**
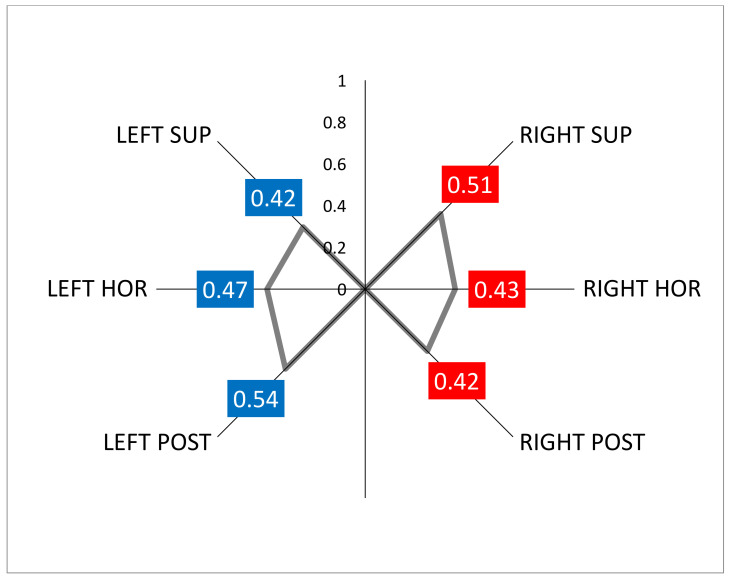
Graphical representation of the mean VOR gain in all patients tested with the vHIT.

The cause of BVL was idiopathic in 11 patients, bilateral Meniere’s disease in 6, bilateral vestibular neuritis in 3, systemic ototoxicity due to aminoglycosides in 2, and CANVAS in 1. In 20 patients we have the vHIT data and the mean gain of the VOR for the six canals is shown in Figure 5. BPPV was idiopathic in all cases and not related to antecedent ear infection or traumatism.

The affected side with BPPV was the right in 13 (56.5%) patients and the left in 10 (43.5%). The affected canal was the posterior in 16 (69.6%) patients, the horizontal in 5 (21.8%) patients, the superior in 1 (4.3%), and, multiple canals in another 1 (4.3%) patient. According to physiopathology 20 were due to canalithiasis and 3 (all of them horizontal canals) to cupulolithiasis. In patients with BPPV, the mean gain of the VOR in the affected canal was 0.52 ± 0.16 while in the corresponding co-planar (and other ear) canal was 0.44 ± 0.23; differences were not significant (*p* > 0.05).

The specific PRM was performed immediately after diagnosis. The number of treatments performed to achieve a negative diagnostic test was only 1 in 13 patients; 1 additional PRM was performed in 5 patients and the rest, 5, needed 2.9 ± 1.1 PRM. There was an association between the initial diagnosis and recurrence such that in 8/10 of those first diagnosed with BPPV and then subsequently of BVL there was at least 1 recurrence that needed a new PRM. Recurrences were significantly lower when the first diagnosis was BVL or both (BVL and BPPV) simultaneous (*p* = 0.006).

In 18/23 patients once the diagnosis of BVL was done vestibular rehabilitation was initiated and all of them considered that to be beneficial.

At one of the centers, the vHIT was performed on all patients diagnosed with BPPV. They were 405 patients: the vHIT was considered abnormal in 86 (21.2%) but only in 6 (1.48%) a diagnosis of BVL was done.

## 4. Discussion

In this work we were interested in analyzing an *a priori* paradoxical situation occasionally found in clinical work as one of them, BVL, should mean the other was impossible, that is, BPPV, by making the vestibular receptor insensitive to any otoconial debris freely floating in the canal or adhered to the cupula. This review was prompted when examining a patient with a recent onset BPPV who was previously diagnosed with BVL after treatment with non-monitored systemic gentamicin due to renal disease. This is not an impossible situation but is infrequent as it represents 1% of those with BPPV (in the prospective search that number was 1.8%) and 10% of those with BVL.

This finding firstly alerts on the need to perform a complete vestibular examination, in other words, the head impulse test (both at the bedside or in clinical form or with technology such as the vHIT) must be part of the vestibular examination in case of a suspected BPPV, and the Dix Hallpike test in a case of suspected BVL.

The rationale for the performance of the head-impulse test or its video-based method (the vHIT, here used) in patients with suspected BPPV (a mechanical disease) has been questioned by the results. Previous authors have not found any significant difference in patients with idiopathic BPPV between the gain of the VOR in the affected semicircular canal or in the asymmetry of VOR gain for co-planar canal pairs and, in none of the patients did they find refixation saccades when stimulating the affected canal [24]. On the contrary, in a prospective study the vHIT response (in terms of gain of the VOR) in patients with BPPV affecting the posterior canal was not found to be significantly different from that in normal controls but significantly different from that in the other ear of the same patient; this difference disappeared one-month after proper PRM treatment [25]. Both results provide two different messages: not to use the vHIT or to do so respectively during a vestibular examination of patients with typical BPPV. Our results are in line with the latest work and when combining the gain of the VOR and saccades registration the number of abnormal results increase in patients tested with the vHIT who were diagnosed with idiopathic BPPV. A recent meta-analysis concluded that otoconial debris could interfere with the normal functioning of the canal ampullae by biasing the cupula in the excitatory or inhibitory direction or by exerting some degree of pressure on the cupula which explains why only posterior semicircular canal BPPV most often displays abnormal VOR values as assessed in the vHIT [26]. 

In the case of BVL, the rationale for the performance of positional testing is well supported by previous works. BPPV has been found commonly among older adults with dizziness, including those not seeking medical care. According to different studies, 25% of these patients (>70 years old) mention unsteadiness or imbalance and not properly vertigo [27]. The resolution of positional nystagmus however does not always follow a significant recovery from unsteadiness.

We have found that BPPV can affect different canals irrespective of the diagnosis of BVL. This suggests that not only the Dix–Hallpike test but complete positional testing should be performed when BVL is suspected in order to verify whether positional nystagmus and vertigo could be part of the problem. The different forms of BPPV found in our work reflect what is found in other neurotology units where posterior canalithiasis is the most frequent finding [5]. In our work we decided not to include the “probable” and “possible” forms of BPPV given the difficulty of clinically dealing with them in the context of patients with BVL; those patients are in a dilemma when trying to reach a definite diagnosis and up to now are on follow-up.

We have found that BPPV and nystagmus occur in cases of moderate vestibular deficit and in our series most probably as coincidental diseases. It is not clear how much vestibular function must be preserved to induce a consistent nystagmus in examination, but, according to our findings, normal vestibular function is not the *sine qua non* for BPPV and nystagmus to occur. Post head-shake nystagmus [28] and skull vibration-induced nystagmus [29] are also closely dependent on the amount of vestibular hypofunction and degree of asymmetry between the normal and affected side; in the case of SVIN in vestibular neuritis when gain asymmetry is >31%, 95% of the patients show SVIN. However, in BPPV, induced nystagmus occurs because of otoconia stimulation of the hypofunctional receptor: the location, number, and size of the particles must also be considered [30]. The vHIT allows for a precise analysis of the VOR and we can say that at least when the gain of the VOR is 0.52, free-floating otoconia in the semicircular canal or attached to the cupula still exert a degree of pressure in the vestibular receptor and provoke a typical nystagmus response. It is a common experience in the final stage of Ménière’s disease that some patients still suffer vertigo episodes, and the caloric test shows a hypofunctional response and even no response at all with ice-water stimulation. The cessation of those crises after surgery indicates the existence of some controversy between clinical symptomatology and vestibular function tests results. In a large study, no correlation was found between disease duration (in years), stage of the disease (by hearing loss), and results in the caloric test and vHIT of the horizontal semicircular canals [31]. When the evaluation is extended to all semicircular canals and the vHIT is used then a trend to abnormal results was found for the vertical canals in that type of patients [32]. Saccular function as measured with the cervical vestibular evoked myogenic potentials (cVEMP) has clearly also shown abnormal results according to stage (as measured by hearing loss) showing a tuning shift to a higher best stimulation frequency [33].

Patients were treated following the usual methodology for each disorder and no specific or different procedure was considered, which also supports the idea of purely coincidental disorders. Taking into consideration the limitations of BPPV treatment in this particular elderly population [34], the results are similar to those shown by others. When both diagnoses were made at the same time (in 9/23 patients), the PRM was performed and, from the beginning, clearly identified the need for a further vestibular rehabilitation program; the treatment of BPPV did not result in a major change to chronic unsteadiness. On the contrary, BPPV was diagnosed in 4/23 patients, while in follow-up for their BVL, because of a major change in unsteadiness which alarmed them enough to come for follow-up, during clinical assessment for the first time they mentioned aggravation while moving the head up or down and getting in or out the bed or rolling to either side. The PRM was found to provide a significant amelioration of symptoms. The patients who were first diagnosed with BPPV and subsequently with BVL were seen because of an exacerbation in symptoms that went from typical spells to chronic unsteadiness which was slightly worse when performing positional changes; all patients (10/23) mentioned the “quality” change in symptoms as not being like those previously treated [35]. We were not able to track exactly when dizziness began, except that was not close to the latest PRM. However, BVL should return a differential diagnosis of residual dizziness, a commonly used term to characterize patients that report imbalance without positional vertigo after PRM treatment and resolution of nystagmus [36,37].

In conclusion, our findings support the need for a comprehensive bedside vestibular examination in patients irrespective of a suspected diagnosis or when the clinical follow-up shows a significant change in any given patient or only slight in the elderly. When BPPV and BVL are seen together, they should be managed as when seen alone. When chronic dizziness is the main clinical manifestation after PRM in patients with BPPV, it is important to consider BVL in the differential diagnosis.

## Figures and Tables

**Table 1:** Diagnostic criteria A and B of bilateral vestibulopathy, according to the consensus document of the Committee for the Classification of Vestibular Disorders of the Bárány Society (2017).

A. Chronic vestibular syndrome with the following symptoms
1. Unsteadiness when walking or standing plus at least one symptom from 2 or 3 2. Movement-induced blurred vision or oscillopsia during walking or quick head/body movements 3. Worsening unsteadiness in darkness and/or on uneven ground
B. No symptoms while sitting or lying down under static conditions

## Data Availability

Data pertaining to this study can be shared upon request to the corresponding author.
